# Boginia virus, a newfound hantavirus harbored by the Eurasian water shrew (*Neomys fodiens*) in Poland

**DOI:** 10.1186/1743-422X-10-160

**Published:** 2013-05-22

**Authors:** Se Hun Gu, Janusz Markowski, Hae Ji Kang, Janusz Hejduk, Beata Sikorska, Paweł P Liberski, Richard Yanagihara

**Affiliations:** 1 Pacific Center for Emerging Infectious Diseases Research, John A. Burns School of Medicine, University of Hawaii at Manoa, 651 Ilalo Street, BSB320L, Honolulu, Hawaii 96813, USA; 2 Department of Teacher Training and Biodiversity Studies, Faculty of Biology and Environmental Protection, University of Łódź, Łódź, Poland; 3 Department of Molecular Pathology and Neuropathology, Medical University of Łódź, Łódź, Poland

**Keywords:** Hantavirus, Neomys, Sorex, Shrew, Phylogeny, Poland

## Abstract

**Background:**

Guided by decades-old reports of hantaviral antigens in the Eurasian common shrew (*Sorex araneus*) and the Eurasian water shrew (*Neomys fodiens*) in European Russia, we employed RT-PCR to analyze lung tissues of soricine shrews, captured in Boginia, Huta Dłutowska and Kurowice in central Poland during September 2010, 2011 and 2012.

**Findings:**

In addition to Seewis virus (SWSV), which had been previously found in Eurasian common shrews elsewhere in Europe, a genetically distinct hantavirus, designated Boginia virus (BOGV), was detected in Eurasian water shrews captured in each of the three villages. Phylogenetic analysis, using maximum likelihood and Bayesian methods, showed that BOGV formed a separate lineage distantly related to SWSV.

**Conclusions:**

Although the pathogenic potential of BOGV and other recently identified shrew-borne hantaviruses is still unknown, clinicians should be vigilant for unusual febrile diseases and clinical syndromes occurring among individuals reporting exposures to shrews.

## Findings

Although hantaviral antigens were detected by the indirect immunofluorescent antibody test and enzyme immunoassay in tissues of the Eurasian common shrew (*Sorex araneus*), Eurasian pygmy shrew (*Sorex minutus*) and Eurasian water shrew (*Neomys fodiens*) in European Russia and the former Yugoslavia more than two decades ago [1-3], shrews (order Soricomorpha) had been dismissed as being unimportant in the ecology and evolution of hantaviruses. Guided by these long-ignored reports, and aided by access to archival tissue collections, an opportunistic search, employing reverse transcription-polymerase chain reaction (RT-PCR), demonstrated a genetically distinct hantavirus, named Seewis virus (SWSV), in a Eurasian common shrew captured in Graubünden, Switzerland [4].

Subsequent studies have indicated that SWSV is widespread across the vast distribution of its soricid reservoir, in Austria, Czech Republic, Slovakia, Finland, Germany, Hungary and Russia [5-7]. SWSV has also been detected in the Siberian large-toothed shrew (*Sorex daphaenodon*) and tundra shrew (*Sorex tundrensis*) in Siberia [6]. And in a comprehensive study on the phylogeography of SWSV in central Europe, SWSV was shown to exhibit distinct geographic-specific clustering in Eurasian common shrews and Eurasian pygmy shrews [7]. The overlapping geographic ranges of the Eurasian common shrew and other shrew species in central Poland provided an opportunity to investigate the existence of SWSV and other soricine shrew-borne hantaviruses.

**Figure 1:**
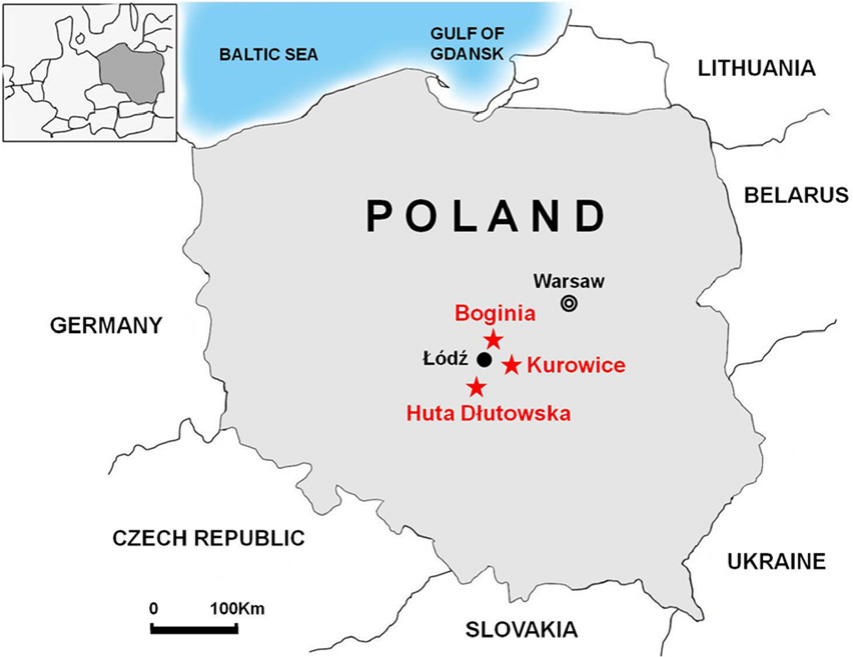
Map of Poland, showing the three villages of Boginia, Huta Dłutowska and Kurowice, in close proximity to Łódź, where hantavirus-infected soricine shrews were captured.

Shrews were captured, using live traps and pitfall traps, placed 5 m apart and baited with raw bacon or beef, during the month of September in 2010, 2011 and/or 2012 in Boginia (51°20′26.80 N, 19°36′41.36 E), Huta Dłutowska (51°35′49.51 N, 19°22′46.80 E) and/or Kurowice (51°39′48.03 N, 19°42′20.92 E), three closely located villages within Łódź East County, in central Poland (Figure 1). All trapping and experimental procedures on shrews were approved by the General Directorate for Environmental Protection (DOP-OZGiZ.4200/N2732/10/JRO, DOP-OZGiZ.6401.05.25.2011kp.3 and DOP-OZGiZ.6401.05.28.2011kp.1).

Total RNA, extracted from lung tissues preserved in RNAlater® RNA Stabilization Reagent (Qiagen) from 40 shrews (Table 1), was analyzed for hantavirus RNA by RT-PCR, using oligonucleotide primers designed from soricomorph-borne hantaviruses [5,8-10]. Using Clustal W [11], pair-wise alignment and comparison of the 795- and 782-nucleotide regions of the M and L segments, respectively, revealed a genetically distinct hantavirus, named Boginia virus (BOGV), in 3 of 6 Eurasian water shrews captured in Boginia, Huta Dłutowska and Kurowice (Tables 1 and 2). The high prevalence of BOGV infection in Eurasian water shrews captured in three separate villages in central Poland suggested a well-established reservoir host-hantavirus relationship.

**Table 1:** RT-PCR detection of hantavirus RNA in tissues of soricine shrews from Poland

**Genus species**	**Capture site**	**Capture dates**	**Number tested**	**Number positive**
*Sorex araneus*	Boginia	September 20–22, 2010	3	1
		September 20–22, 2011	6	0
	Huta Dłutowska	September 15–18, 2011	9	2
*Sorex minutus*	Boginia	September 21–22, 2011	2	0
	Huta Dłutowska	September 16–18, 2011	4	0
	Kurowice	September 7–14, 2012	10	1
*Neomys fodiens*	Boginia	September 22, 2011	1	1
	Huta Dłutowska	September 22, 2011	1	1
	Kurowice	September 9–14, 2012	4	1

**Table 2:** Partial S-, M- and/or L-segment sequences of hantaviruses detected in lung tissues of soricine shrews captured in central Poland

			**Nucleotides and GenBank accession numbers**		
**Virus strain**	**Capture site**	**Capture date**	**S segment**	**M segment**	**L segment**
SWSV 1107	Boginia	22-Sep-2010	690 nt (435–1025) JX990921	647 nt (1520–2100) JX990967	806 nt (2520–3400) JX990936
SWSV 2048	Huta Dłutowska	16-Sep-2011			756 nt (2541–3296) JX990944
SWSV 2049	Huta Dłutowska	17-Sep-2011			357 nt (2900–3300) JX990945
SWSV 2121	Kurowice	12-Sep-2012			316 nt (2998–3313) KC537794
BOGV 2073	Huta Dłutowska	22-Sep-2011			394 nt (2520–2935) JX990964
BOGV 2074	Boginia	22-Sep-2011		795 nt (1520–2355) JX990966	783 nt (2541–3323) JX990965
BOGV 2177	Kurowice	9-Sep-2012			408 nt (2520–2935) KC537795

Abbreviations: BOGV, Boginia virus; SWSV, Seewis virus; nt, nucleotides.

The L-segment genetic variation between BOGV strains 2073, 2074 and 2177 was 3.1–15.0% at the nucleotide level, but there was 100% amino acid homology. The observation that hantaviruses exhibit high sequence conservation at the amino acid level, despite considerable divergence of more than 15% at the nucleotide level, has been made previously [5-7,12]. This has been attributed to the long-standing co-adaptation between virus and reservoir host, as well as the strong selection pressure to preserve function of the three gene products.

BOGV and other representative soricine shrew-borne hantaviruses exhibited M- and L-segment sequence similarity of only 71.7–76.7% and 75.1–87.7% at the nucleotide and amino acid levels, respectively. The taxonomic identities of the BOGV-infected Eurasian water shrews were verified by mitochondrial DNA sequence analysis (GenBank accession no. KC537796 for BOGV 2073; KC537797 for BOGV 2074; and KC537798 for BOGV 2177).

Eurasian common shrews, captured in Boginia and Huta Dłutowska, as well as a Eurasian pygmy shrew from Kurowice, were infected with SWSV (Table 1), indicating simultaneous circulation of genetically distinct hantaviruses in syntopic shrew species captured in the same trap sites. Recently, SWSV was detected in Eurasian pygmy shrews in Germany and the Czech Republic [7], but the low prevalence is suggestive of spillover. Nevertheless, carriage of the same hantavirus in two soricine shrew species in central Poland parallels findings of host sharing of SWSV in other *Sorex* shrew species [6,7], as well as of rodent-borne hantaviruses, such as Tula virus, in several arvicolid rodent species [12-14].

**Figure 2:**
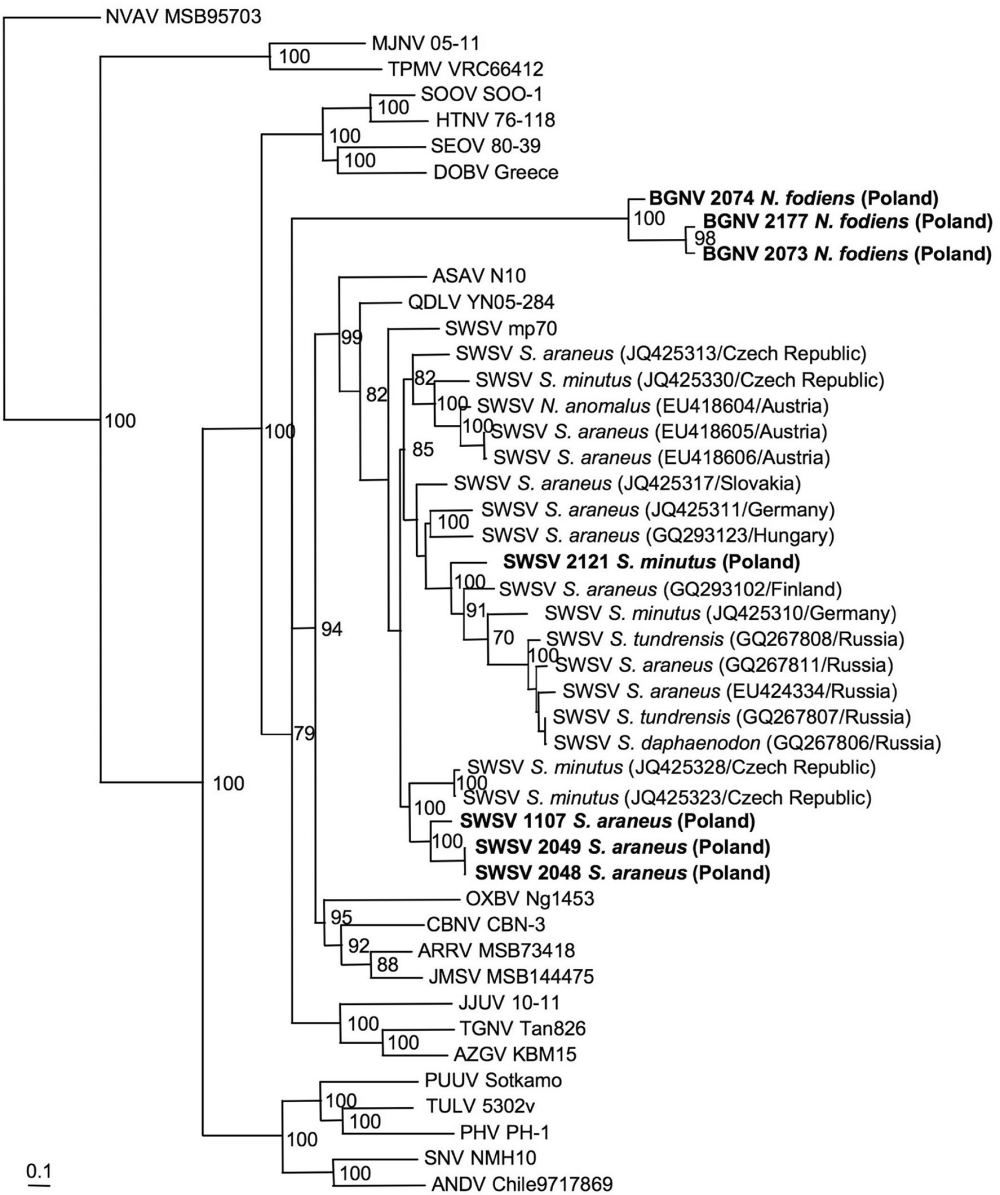
**Phylogenetic tree generated by the maximum-likelihood method, using the GTR + I+Γ model of evolution, based on the alignment of L-segment sequences of BOGV strains 2073 (JX990964), 2074 (JX990965) and 2177 (KC537795).** The phylogenetic positions of BOGV are shown in relationship to representative soricine shrew-borne hantaviruses, including Cao Bang virus (CBNV CBN-3, EF543525), Ash River virus (ARRV MSB73418, EF619961), Jemez Springs virus (JMSV MSB144475, FJ593501) and Qiandao Lake virus (QDLV YN05-284, GU566021). Representative strains of Seewis virus (SWSV) from Poland and other widely separated geographic regions are indicated by their country of origin and GenBank numbers. Also shown are crocidurine shrew-borne hantaviruses, including Thottapalayam virus (TPMV VRC66412, EU001330), Imjin virus (MJNV 05–11, EF641806), Tanganya virus (TGNV Tan826, EF050454), Azagny virus (AZGV KBM15, JF276228) and Jeju virus (JJUV 10–11, HQ834697); and mole-borne hantaviruses, including Asama virus (ASAV N10, EU929078), Nova virus (NVAV MSB95703, FJ593498) and Oxbow virus (OXBV Ng1453, FJ593497). Rodent-borne hantaviruses include Hantaan virus (HTNV 76–118, NC_005222), Soochong virus (SOOV SOO-1, DQ056292), Dobrava virus (DOBV Greece, NC_005235), Seoul virus (SEOV 80–39, NC_005238), Tula virus (TULV M5302v, NC_005226), Puumala virus (PUUV Sotkamo, NC_005225), Prospect Hill virus (PHV PH-1, EF646763), Sin Nombre virus (SNV NMH10, NC_005217) and Andes virus (ANDV Chile9717869, AF291704). The numbers at each node are bootstrap values, expressed as percentages, based on 1,000 iterations, and the scale bar indicates nucleotide substitutions per site.

Phylogenetic trees, generated by maximum likelihood and Bayesian methods, implemented in PAUP* (Phylogenetic Analysis Using Parsimony, 4.0b10) [15], RAxML Blackbox webserver [16] and MrBayes 3.1 [17], under the best-fit GTR + I + Γ model of evolution established using jModeltest 0.1.1 [18], showed that BOGV formed a separate lineage distantly related to SWSV (Figure 2), in keeping with the evolutionary relationship of their soricid hosts. As expected, phylogenetic trees showed SWSV to segregate generally along geographic-specific lineages (Figure 2), as reported previously [5-7].

As for several other shrew-borne hantaviruses, the sequence divergence of BOGV presented insurmountable difficulties in obtaining the full genome sequence. Sequencing efforts were also severely constrained by the limited availability of tissues, and poor-quality tissue RNA thwarted efforts at employing next generation sequencing technology. Efforts, now underway, to rapidly freeze tissues collected from Eurasian water shrews captured in central Poland will be used for virus isolation attempts.

The detection of a novel, genetically distinct hantavirus in the Eurasian water shrew captured in central Poland confirms decades-old reports that this shrew species might serve as a reservoir [2]. Whether other *Neomys* species, such as the Mediterranean water shrew (*Neomys anomalus*), also harbor a BOGV-like hantavirus, rather than SWSV, is unknown. Nevertheless, neither physical proximity with sharing of habitats nor genetic relatedness of shrew host species allows accurate prediction of the hantavirus species in a particular reservoir. That said, this study provides clear evidence of the co-existence of two genetically distinct hantaviruses in their soricomorph reservoir host species inhabiting the same ecological niche. To what extent hantavirus spillover occurs between reservoir shrews and rodents is currently under investigation.

Unlike most soricine shrew species, the Eurasian water shrew is comparatively large, measuring approximately 10 cm in body length, with a long tail. It is highly territorial and lives a solitary life near freshwater and other wetland habitats, including rivers, streams, marshes, bogs and damp grasslands and meadows, at sea level to elevations over 2,500 m [19,20]. Eurasian water shrews forage almost exclusively underwater, using to great effect their venomous saliva to feast on aquatic invertebrates, including insects, gastropods and crustaceans, as well as occasionally small vertebrates, such as amphibians and fish. Some terrestrial insects, such as dipteran larvae, are also consumed. The vast distribution of the Eurasian water shrew [19,20], spanning from the United Kingdom and Scandinavia throughout Europe and much of Asia, including far eastern Russia and Sakhalin, where other syntopic soricomorphs and rodents reside, provides rich opportunities to investigate the genetic diversity and phylogeography of BOGV.

That distinct diseases or clinical syndromes have yet to be attributed to shrew-borne hantaviruses is not particularly surprising, given that most hantaviruses are nonpathogenic. Neotomine and sigmodontine rodents were known to harbor hantaviruses long before the terrifying outbreak of a rapidly progressive, frequently fatal disease (now known as hantavirus cardiopulmonary syndrome) signaled the highly pathogenic nature of these hantaviruses [21-23]. Similarly, while the pathogenic potential of BOGV and other recently identified, still-orphan soricid-borne hantaviruses is still unknown, active vigilance by physicians and public health workers for unusual febrile syndromes, occurring among individuals reporting either contact with shrews or exposure to wetlands that may be contaminated by the Eurasian water shrew, would be crucial to uncover an etiological association. Intensive investigations are underway to isolate and further characterize BOGV to ascertain its impact on human health.

## Competing interests

The authors declare that they have no competing interests.

## Authors’ contributions

SHG and HJK performed primer design, RNA extraction, RT-PCR and DNA sequencing reactions and phylogenetic analysis. JM and JH conducted trapping expeditions and collected shrew tissues. BS and PPL provided logistical support. RY conceived the project and provided overall scientific oversight. All authors contributed to the preparation of the final manuscript. All authors read and approved the final manuscript.

## References

[B1] Gavrilovskaya IN Apekina NS Myasnikov YA Bernshtein AD Ryltseva EV Gorbachkova EA Chumakov MP Features of circulation of hemorrhagic fever with renal syndrome (HFRS) virus among small mammals in the European U.S.S.R Arch Virol 1983 75 313 316 10.1007/BF01314898 6220688

[B2] Tkachenko EA Ivanov AP Donets MA Miasnikov YA Ryltseva EV Gaponova LK Bashkirtsev VN Okulova NM Drozdov SG Slonova RA Somov GP Astakhova TI Potential reservoir and vectors of haemorrhagic fever with renal syndrome (HFRS) in the U.S.S.R Ann Soc Belg Med Trop 1983 63 267 269 6229222

[B3] Gligic A Stojanovic R Obradovic M Hlaca D Dimkovic N Diglisic G Lukac V Ler Z Bogdanovic R Antonijevic B Ropac D Avsic T LeDuc JW Ksiazek T Yanagihara R Gajdusek DC Hemorrhagic fever with renal syndrome in Yugoslavia: epidemiologic and epizootiologic features of a nationwide outbreak in 1989 Eur J Epidemiol 1992 8 816 825 10.1007/BF00145326 1363468

[B4] Song J-W Gu SH Bennett SN Arai S Puorger M Hilbe M Yanagihara R Seewis virus, a genetically distinct hantavirus in the Eurasian common shrew (*Sorex araneus*) Virol J 2007 4 114 10.1186/1743-422X-4-114 17967200 PMC2186316

[B5] Kang HJ Arai S Hope AG Song J-W Cook JA Yanagihara R Genetic diversity and phylogeography of Seewis virus in the Eurasian common shrew in Finland and Hungary Virol J 2009 6 208 10.1186/1743-422X-6-208 19930716 PMC2789066

[B6] Yashina LN Abramov SA Gutorov VV Dupal TA Krivopalov AV Panov VV Danchinova G Vinogradov V Luchnikova E Hay J Kang HJ Yanagihara R Seewis virus: phylogeography of a shrew-borne hantavirus in Siberia, Russia Vector Borne Zoonotic Dis 2010 10 585 591 10.1089/vbz.2009.0154 20426688 PMC2979336

[B7] Schlegel M Radosa L Rosenfeld UM Schmidt S Triebenbacher C Löhr PW Fuchs D Heroldová M Jánová E Stanko M Mošanský L Fričová J Pejčoch M Suchomel J Purchart L Groschup MH Krüger DH Klempa B Ulrich RG Broad geographical distribution and high genetic diversity of shrew-borne Seewis hantavirus in Central Europe Virus Genes 2012 45 48 55 10.1007/s11262-012-0736-7 22467179

[B8] Song J-W Kang HJ Song KJ Truong TT Bennett SN Arai S Truong NU Yanagihara R Newfound hantavirus in Chinese mole shrew, Vietnam Emerg Infect Dis 2007 13 1784 1787 10.3201/eid1311.070492 18217572 PMC2262106

[B9] Arai S Ohdachi SD Asakawa M Kang HJ Mocz G Arikawa J Okabe N Yanagihara R Molecular phylogeny of a newfound hantavirus in the Japanese shrew mole (*Urotrichus talpoides*) Proc Natl Acad Sci USA 2008 105 16296 16301 10.1073/pnas.0808942105 18854415 PMC2567236

[B10] Kang HJ Bennett SN Sumibcay L Arai S Hope AG Mocz G Song J-W Cook JA Yanagihara R Evolutionary insights from a genetically divergent hantavirus harbored by the European common mole (*Talpa europaea*) PLoS One 2009 4 e6149 10.1371/journal.pone.0006149 19582155 PMC2702001

[B11] Thompson JD Higgins DG Gibson TJ CLUSTAL W: improving the sensitivity of progressive multiple sequence alignment through sequence weighting, position-specific gap penalties and weight matrix choice Nucleic Acids Res 1994 22 4673 4680 10.1093/nar/22.22.4673 7984417 PMC308517

[B12] Schlegel M Kindler E Essbauer SS Wolf R Thiel J Groschup MH Heckel G Oehme RM Ulrich RG Tula virus infections in the Eurasian water vole in Central Europe Vector Borne Zoonotic Dis 2012 12 503 513 10.1089/vbz.2011.0784 22225425

[B13] Schmidt-Chanasit J Essbauer S Petraityte R Yoshimatsu K Tackmann K Conraths FJ Sasnauskas K Arikawa J Thomas A Pfeffer M Schaminghausen JJ Spiettstoesser W Wenk M Heckel G Ulrich RG Extensive host sharing of central European Tula virus J Virol 2010 84 459 474 10.1128/JVI.01226-09 19889769 PMC2798396

[B14] Song J-W Gligic A Yanagihara R Identification of Tula hantavirus in *Pitymys subterraneus* captured in the Cacak region of Serbia-Yugoslavia Int J Infect Dis 2002 6 31 36 10.1016/S1201-9712(02)90133-5 12044299

[B15] Swofford D PAUP*: Phylogenetic Analysis Using Parsimony (*and Other Methods) 2003 Sunderland, Massachusetts: Sinauer Associates

[B16] Stamatakis A Hoover P Rougemont J A rapid bootstrap algorithm for the RAxML web servers Syst Biol 2008 57 758 771 10.1080/10635150802429642 18853362

[B17] Ronquist F Huelsenbeck JP MrBayes 3: Bayesian phylogenetic inference under mixed models Bioinformatics 2003 19 1572 1574 10.1093/bioinformatics/btg180 12912839

[B18] Posada D jModelTest: phylogenetic model averaging Mol Biol Evol 2008 25 1253 1256 10.1093/molbev/msn083 18397919

[B19] Nowak RM Walker’s Mammals of the World 1999 5 Baltimore, Maryland: Johns Hopkins University Press

[B20] Spitzenberger F Mitchell-Jones AJ, Amori G, Bogdanowicz W, Krystufek B, Reijnders PHJ, Spitzenberger F, Stubbe M, Thissen JBM, Vohralík V, Zima J Neomys fodiens The Atlas of European Mammals. T & AD Poyser for the Societas Europaea Mammalogica 1999 London: Academic Press 61 62

[B21] Yanagihara R Daum CA Lee P-W Baek LJ Amyx HL Gajdusek DC Gibbs CJ Jr Serological survey of prospect hill virus infection in indigenous wild rodents in the USA Trans R Soc Trop Med Hyg 1987 81 42 45 10.1016/0035-9203(87)90275-6 2895510

[B22] Nichol ST Spiropoulou CF Morzunov S Rollin PE Ksiazek TG Feldmann H Sanchez A Childs J Zaki S Peters CJ Genetic identification of a hantavirus associated with an outbreak of acute respiratory illness Science 1993 262 914 917 10.1126/science.8235615 8235615

[B23] Nerurkar VR Song J-W Song K-J Nagle JW Hjelle B Jenison S Yanagihara R Genetic evidence for a hantavirus enzootic in deer mice (*Peromyscus maniculatus*) captured a decade before the recognition of hantavirus pulmonary syndrome Virology 1994 204 563 568 10.1006/viro.1994.1570 7941323

